# Myeloid transformation of plasma cell myeloma: molecular evidence of clonal evolution revealed by next generation sequencing

**DOI:** 10.1186/s13000-018-0692-1

**Published:** 2018-02-20

**Authors:** Jonathon H. Gralewski, Ginell R. Post, Frits van Rhee, Youzhong Yuan

**Affiliations:** 1 Department of Pathology, University of Arkansas for Medical Sciences, Little Rock, AR 72205-7199 USA; 2 Myeloma Institute, University of Arkansas for Medical Sciences, Little Rock, AR USA

**Keywords:** Clonal evolution, Myeloid sarcoma, Molecular profiling, Next generation sequencing, Transdifferentiation, Plasma cell myeloma, RAS/RAF signaling pathway

## Abstract

**Background:**

Plasma cell myeloma (PCM) is a neoplasm of terminally differentiated B lymphocytes with molecular heterogeneity. Although therapy-related myeloid neoplasms are common in plasma cell myeloma patients after chemotherapy, transdifferentiation of plasma cell myeloma into myeloid neoplasms has not been reported in literature. Here we report a very rare case of myeloid neoplasm transformed from plasma cell myeloma.

**Case presentation:**

A 60-year-old man with a history of plasma cell myeloma with IGH-MAF gene rearrangement and RAS/RAF mutations developed multiple soft tissue lesions one year following melphalan-based chemotherapy and autologous stem cell transplant. Morphological and immunohistochemical characterization of the extramedullary disease demonstrated that the tumor cells were derived from the monocyte-macrophage lineage. Next generation sequencing (NGS) studies detected similar clonal aberrations in the diagnostic plasma cell population and post-therapy neoplastic cells, including IGH-MAF rearrangement, multiple genetic mutations in RAS signaling pathway proteins, and loss of tumor suppressor genes. Molecular genetic analysis also revealed unique genomic alterations in the transformed tumor cells, including gain of NF1 and loss of TRAF3.

**Conclusion:**

To our knowledge, this is the first case of myeloid sarcoma transdifferentiated from plasma cell neoplasm. Our findings in this unique case suggest clonal evolution of plasma cell myeloma to myeloma neoplasm and the potential roles of abnormal RAS/RAF signaling pathway in lineage switch or transdifferentiation.

## Background

Plasma cell myeloma (PCM) is a neoplasm of terminally differentiated B lymphocytes and most often involves the bone marrow. Malignant plasma cells are defined immunophenotypically by diminished or increased expression of at least two antigens not present on non-neoplastic plasma cells. For example, malignant plasma cells show decreased or loss of CD45 and/or CD19 expression and may aberrantly express CD56, CD117, and/or CD20 [1]. Immunohistochemical stains performed on diagnostic biopsies demonstrate retained expression of non-neoplastic plasma cell antigens (e.g. CD138 and MUM-1) and cytoplasmic light chain restriction. PCM is characterized by molecular heterogeneity, including balanced translocations involving the immunoglobulin heavy chain locus, complex karyotypes, and mutations in the RAS signaling cascade [2–4]. For example, KRAS, NRAS and BRAF mutations are detected in approximately 33% of newly diagnosed PCM patients [3, 4].

Disease progression in PCM can be associated with disease at extramedullary sites, high grade plasma cell morphology, acquisition of additional genetic mutations or possibly reactivation of Epstein Barr virus infection [3, 5–7]. Although plasma cells may resemble blasts and express aberrant myeloid antigens [5, 8], malignant plasma cells retain expression of a subset of plasma cell markers and demonstrate light chain restriction, allowing immunohistochemical classification of the tumor as PCM. A subset of patients with PCM develop secondary malignancies following high dose chemotherapy. Therapy-related myeloid neoplasms are the most common secondary malignancy in PCM [9]. We recently described the rapid onset of therapy-related acute leukemia in patients in complete remission for PCM. In this series, the immunophenotype and karyotype of the leukemic cells was distinctly different than that seen in the original PCM [10].

Here we report a rare case of multiple soft tissue sarcomas arising in a patient in complete remission for high-risk PCM. Immunohistochemical stains and flow cytometry showed that the tumor cells expressed monocyte-macrophage (CD163, CD68 and lysozyme) and myeloid antigens (myeloperoxidase and CD13) without plasma cell markers. Fluorescence in situ hybridization (FISH) and next generation sequencing (NGS) studies demonstrated a clonal relationship between the diagnostic PCM and transformed tumor cells, including loss of tumor suppressor genes and multiple, clonal/subclonal mutations in the RAS pathway. To our knowledge, this is the first reported case of myeloid transformation in PCM.

## Case presentation

**Fig. 1:**
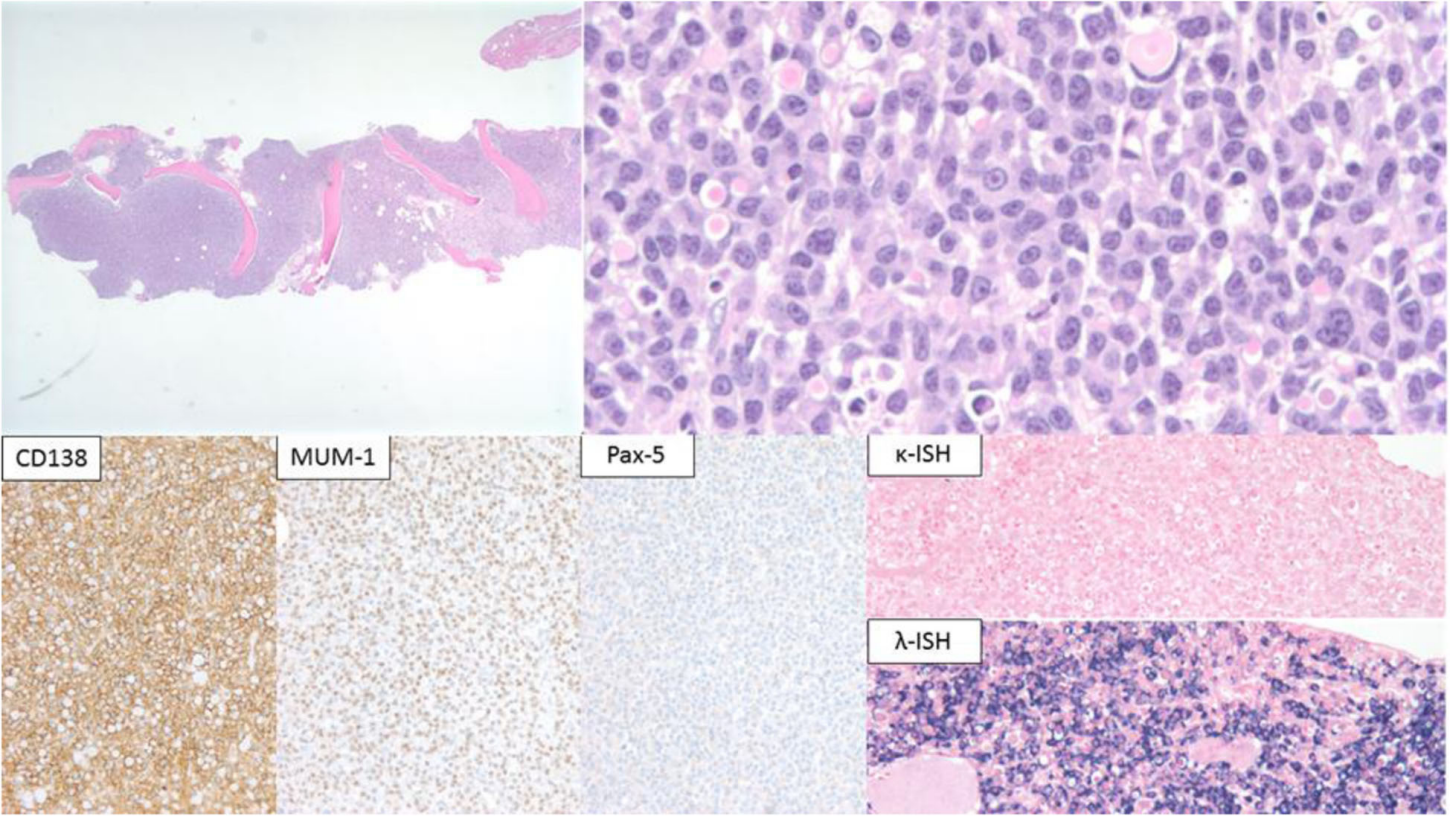
Morphologic examination (top) and immunohistochemical stains (bottom) performed on the diagnostic bone marrow core biopsy. The H&E stained sections of the bone marrow biopsy revealed a diffuse infiltrate of plasma cells (× 20) with nuclear pleomorphism; a subset of plasma cells showed prominent nucleoli (× 500). The plasma cells expressed CD138, MUM-1 with lambda light chain restriction (× 200)

A sixty-year-old Caucasian male presented to our institution with chest pain. A complete blood count showed anemia (hemoglobin of 8.0 g/dL; reference range 13–17 g/dL) and thrombocytopenia (platelet count of 69,000/μL; reference range 150,000–450,000/μL). Review of the peripheral blood smear revealed 12% circulating plasma cells. Additional laboratory evaluation demonstrated an elevated total serum protein of 11.3 g/dL (reference range: 6.4–8.3 g/dL) and serum M-component (6.8 g/dL) with increased IgG (9010 mg/dL; reference range: 700–1600 mg/dL) and free lambda light chain (120 mg/dL; reference range 0.57–2.63 mg/dL). The diagnostic bone marrow biopsy demonstrated a hypercellular bone marrow for age extensively involved by lambda light chain-restricted plasma cells (Fig. 1). Concurrent flow cytometric analysis showed that the neoplastic plasma cells were positive for CD38, CD138, and CD20 (heterogeneous) and negative for CD45, CD27, CD81, CD56, and CD19. Cytogenetic analysis of the bone marrow aspirate cells revealed a complex karyotype: 43,XY,del(1)(p13p32),+ 3,der(3;6)(q10;p10),del(5)(q15q33),? t(9;15)(p24;q24),-10,add(13)(p11.2),del(14)(q24),-20,-22,inc[3]/46,XY[17]. Myeloma FISH studies were uninformative due to paucity of analyzable plasma cells. Gene array studies were consistent with high-risk c-MAF subgroup [2]. Foundation One™ NGS studies revealed IGH-MAF gene rearrangement and several genomic alterations, including BRAF G469V and G466A, KRAS A146V, MAP3K6 Q943, CDKN2A/B loss, TRAF3 R505 and PTPRO E379K (Table 1). Magnetic-resonance imaging (MRI) and positron-emitted topography (PET) scans highlighted multiple focal lesions in the cervical spine, rib cage, tibia, and fibula, but no extramedullary disease.

**Table 1:** Next-generation sequencing of the diagnostic bone marrow aspirate and left gingival lesion

Bone marrow aspirate	Soft tissue
*BRAF1* G466A subclonal, G469 subclonal	*BRAF* G469A
*KRAS* A146V	*KRAS* A146V
*IGH* IGH-MAF rearrangement	*IGH* IGH-MAF rearrangement
*CDKN2A/B* loss	*CDKN2A/B* loss
*MAP3K6* Q943, truncation exon 22	*MAP3K6* Q943, truncation exon 22
*TRAF3* R505	*NF1* R2450
*PTPRO* E379K – subclonal	*CCT6B* splice site 615-2A > G *TNFAIP3* W85

**Fig. 2:**
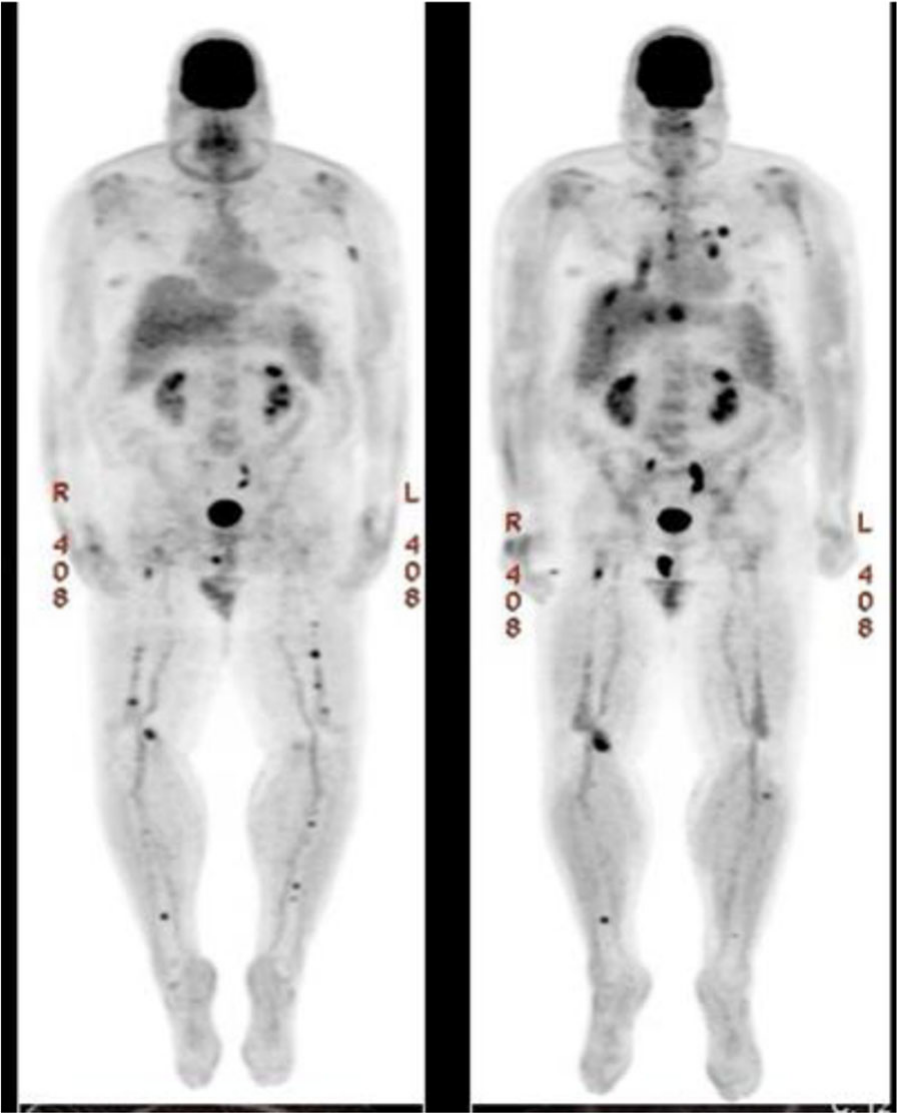
PET-CT image (left) showing the lesions in the proximal right tibia, right proximal femur and perineum. PET-CT image (right) demonstrate increased size of the previous lesions and new lesions in the left mandibular soft tissue, liver, lungs and mediastinum three months after first time PET-CT

The patient received induction chemotherapy (Velcade, Dexamethasone, Thalidomide-Cisplatin, Doxorubicin, Cyclophosphamide and Etoposide; VDT-PACE) followed by cytoreduction (Cytoxan, Etoposide, Mesna, Cisplatin, Dexamethasone and Cytarabine; PACMED) and bridging therapy with carfilzomib and daratumumab. An autologous stem cell transplant was performed 10 months after initial diagnosis. Two months after stem cell transplant, bone marrow evaluation was morphologically negative for PCM with no minimal residual disease detected by 8-color flow cytometry; however, PET-CT imaging showed multiple focal lesions in the bilateral femoral shafts, humeri and a 1.8 × 1.2 cm mass in the right perineal region (Fig. 2). A PET-CT imaging study showed that the lesion in the perineal region had increased in size to approximately 3.1 × 2.1 cm with new extramedullary lesions noted in the left mandibular soft tissue, lungs/mediastinal lymph nodes and liver (Fig. 2). The differential diagnosis included multifocal myelomatous disease progression versus infectious etiology. The patient underwent fine needle aspiration of mediastinal lymph nodes and punch biopsy of the gingival lesion.

**Fig. 3:**
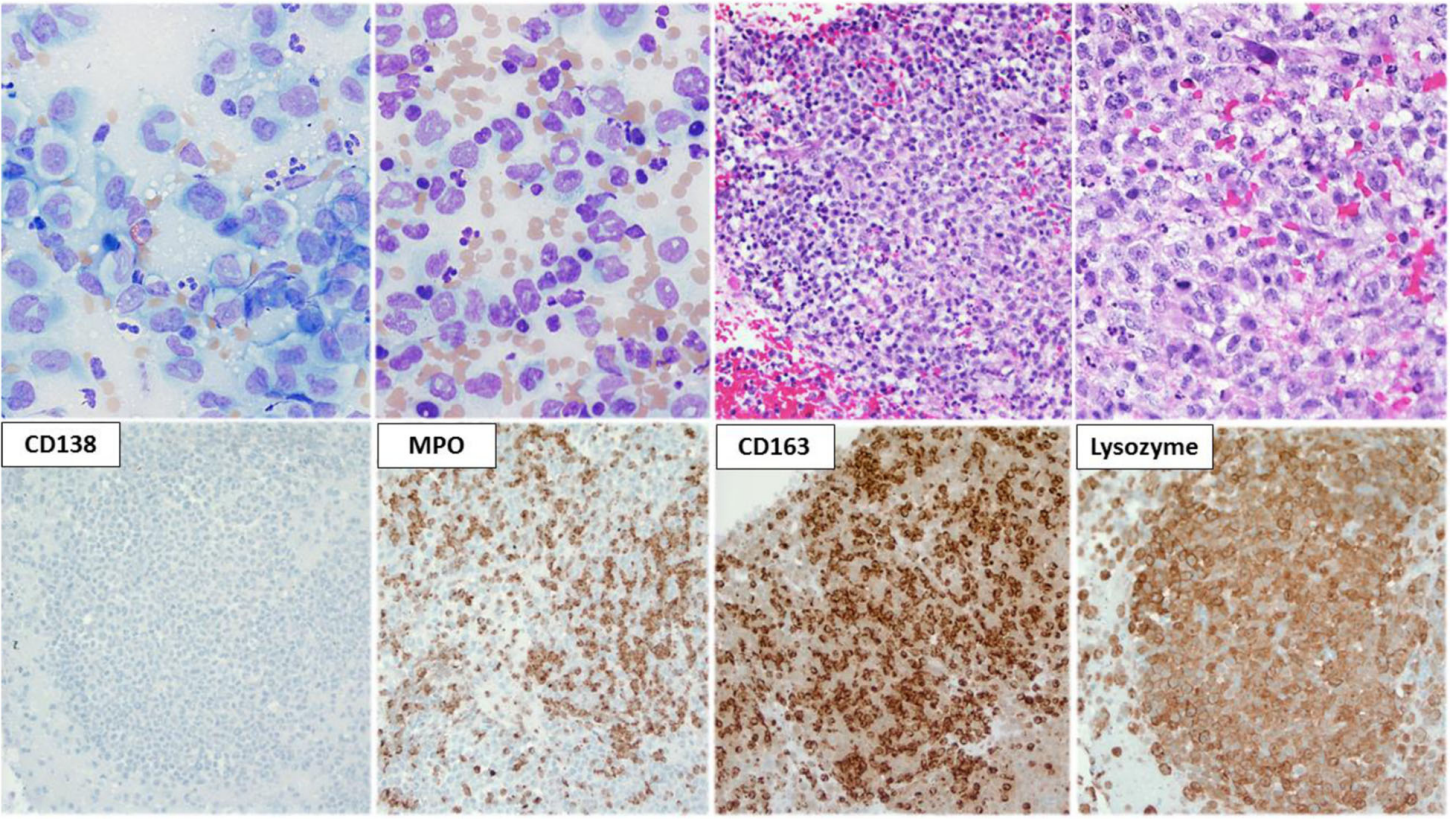
Morphologic examination and immunohistochemical stains performed on the fine needle aspirate of the mediastinal lymph node. The Diff-Quik™ stained slides show large monocytoid cells with ample blue-grey cytoplasm, round to irregular nuclear contours and immature chromatin (× 500, top-left). The H&E stained sections prepared from the cell block shows a diffuse infiltrate of monocytoid cells with ample cytoplasm and round to irregular nuclear contours (× 200 and × 400, top-right). The tumor cells were negative for CD138 and positive for myeloperoxidase (MPO), CD163 and lysozyme (× 200)

**Fig. 4:**
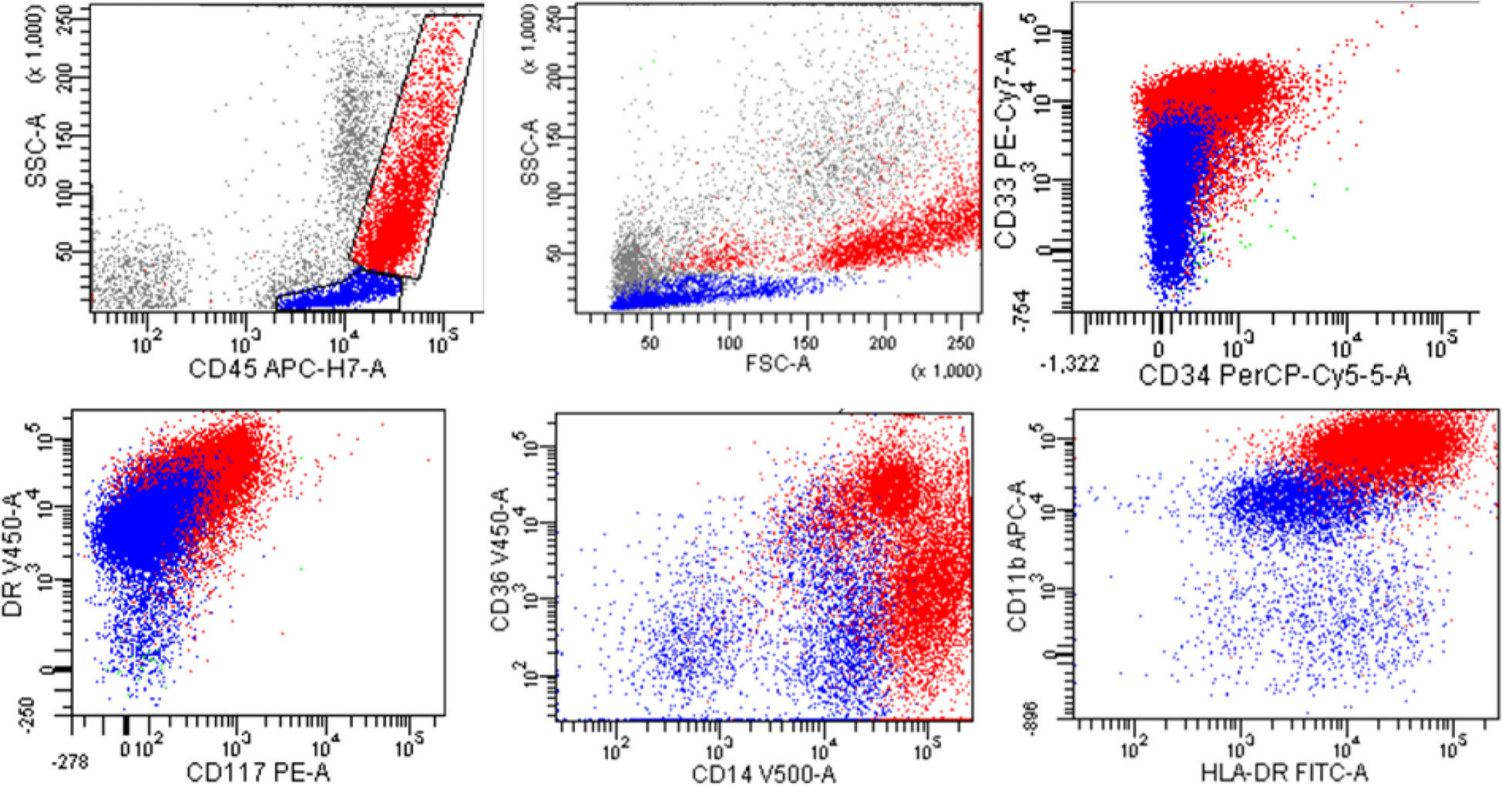
Flow cytometric analysis of mediastinal lymph node revealed two cell populations with distinct forward and side scatter and CD45 expression intensity. One population with increased side and forward scatter comprised 40% of total events (red). This population expressed CD33, HLA-DR, CD36 (variable), CD14 (bright) and CD11b (bright) and were negative for CD34 and CD117. A second population of cells with decreased forward and side scatter showed a similar immunophenotype with variable expression of CD33 and dimmer CD11b and CD14 (blue). These cells were also negative for CD34 and CD117

**Fig. 5:**
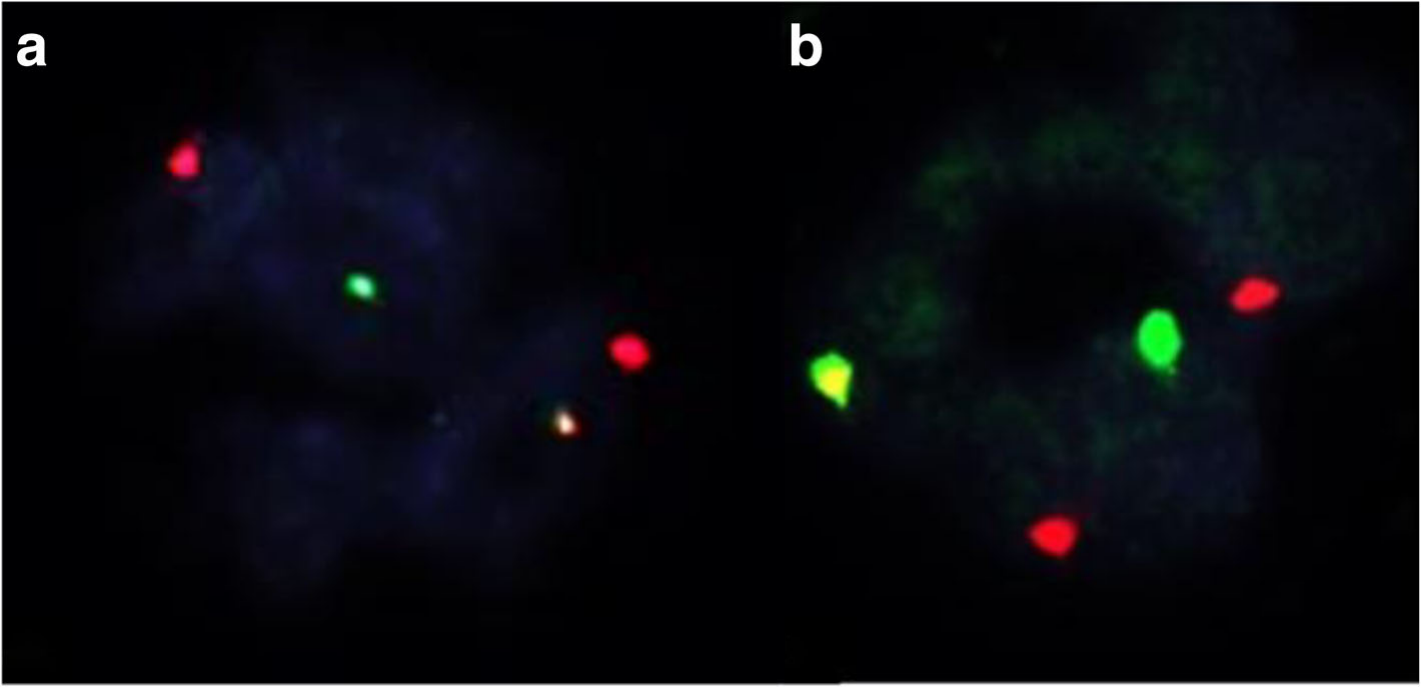
Fluorescence in-situ hybridization studies demonstrating t(14;16)(q32;q23) in both mediastinal lymph node (**a**) and gingival biopsy (**b**). The abnormal signaling patterns are 2R1G1F (**a**) and 2R1G1F (**b**) by dual color, dual fusion probes

The Diff-Quik™ stained sections prepared from the mediastinal lymph node FNA showed large atypical cells with abundant cytoplasm with immature chromatin (Fig. 3). The H&E stained sections prepared from the cell block demonstrated similar morphologic features including an infiltrate of immature monocytic cells with rare mature granulocytes (Fig. 3). Immunohistochemical stains showed that the neoplastic cells expressed myeloperoxidase (MPO; subset), CD163, lysozyme, and were negative for CD138 (Fig. 3). Additional immunohistochemical studies revealed positivity for CD68 and lack of MUM-1, PAX-5, CD56, S-100, and P53 expression (not shown). Concurrent flow cytometric analysis revealed atypical cell populations with distinct CD45 expression and forward and side scatter properties comprising 80% of total analyzed events. One population with increased side and forward scatter comprised 40% of total events. These cells expressed CD45 (bright), CD33, HLA-DR, CD14 (bright), CD11b (bright) and CD36 (variable) (Fig. 4; red), consistent with monocytic lineage. A second population of cells with decreased forward and side scatter showed a similar immunophenotype with variable expression of CD33 and dimmer expression of CD45, CD11b and CD14 (Fig. 4; blue). Both populations were negative for CD34 and CD117. The H&E stained sections of the gingival biopsy showed similar morphologic features, including a dermal infiltrate of large, immature cells with irregular nuclear contours and ample cytoplasm (not shown). Immunohistochemical stains of the gingival biopsy showed an immunophenotype similar to the mediastinal lymph node, including CD68, CD163, lysozyme, MPO (subset) expression and lack of CD138, MUM-1, PAX-5, CD34, and CD56 expression (not shown). FISH studies performed on the mediastinal lymph node and gingival biopsies revealed a translocation between chromosomes 14 and 16 [IGH and MAF genes; t(14;16)(q32;q23)] in approximately 76% and 73% of interphase nuclei examined (Fig. 5), respectively. FISH probes for t(11q23) and del(17p13.1) showed normal signal patterns. Cytogenetic studies performed on the mediastinal lymph node were unsuccessful due to no cell growth. The gingival lesion showed a normal karyotype (46,XY[4]/45,Y,-X[1]) with a low mitotic index. NGS studies performed on the gingival lesion demonstrated IGH-MAF rearrangement, BRAF and KRAS mutations, CDKN2A/B loss, TNFAIP3 and NF1 mutations (Table 1).

The patient received induction chemotherapy (7 + 3 regimen of cytarabine and idarubicin). At follow up, the patient developed neutropenic fever with persistent pancytopenia despite receiving recombinant granulocytic colony stimulating factor. He was re-admitted one week later for possible sepsis/bacteremia. As the patient’s health continued to deteriorate and the soft tissue masses continued to grow, he decided to stop treatments and medical interventions. The patient went into palliative care approximately two months after the diagnosis of multifocal myeloid sarcoma and died shortly thereafter.

## Discussion

We report a case of multiple soft tissue sarcomas in a patient in complete remission for high-risk PCM. The neoplastic cells at the extramedullary sites were large with immature chromatin and expressed the monocyte-macrophage antigen CD163 [11], CD14 and CD68 as well as myeloid antigens MPO and CD13. The tumor cells were negative for S-100 and CD138, consistent with absence of dendritic cell differentiation [12, 13] or relapsed PCM, respectively. The immature nuclear features and expression of the myeloid antigens MPO and CD13 was most consistent with the diagnosis of myeloid sarcoma with monocytic differentiation. Furthermore, FISH studies showed IGH-MAF translocation in the transformed cells and PCR identified an IGH gene rearrangement. NGS- based assays revealed similar genomic alterations suggesting a clonal relationship between the original PCM and secondary myeloid sarcoma.

Hematopoiesis, once viewed as a unidirectional maturation of pluripotent hematopoietic stem cells into specific lineages (such as lymphoid and myeloid), shows considerable plasticity in both normal and malignant hematopoietic cells. Lineage switching has been described in histiocytic sarcoma (HS), Langerhans cell sarcoma or dendritic cell tumor that occur secondary to or synchronous with mediastinal germ cell tumors [14], lymphoid [12, 13, 15–22], and myeloid malignancies [23, 24]. Histiocytic sarcoma cells are derived from bone marrow monocyte precursors [25], expresses monocyte-macrophage antigens (CD163, CD68, and lysozyme) and lack expression of myeloid antigens such as CD33, CD13 and MPO [11, 25, 26]. The identification of clonal associations between HS and antecedent malignancies suggests that HS or myeloid sarcoma can differentiate from other cell lineages or develop from a common progenitor cell [12–20, 23, 27].

Several studies indicate that commitment to specific lineage and lineage conversion depends on the activity of lineage-specific transcription factors [28–30]. For example, PAX-5 is required for B cell differentiation and commitment to B cell lineage [31–33]. B cells that lack PAX-5 expression can differentiate into macrophages, dendritic cells, osteoclasts, granulocytes and natural killer cells [32]. Similarly, the activity of the transcription factors PU.1 and CCAAT/enhancer binding protein alpha (C/EBPα) are important for myeloid progenitor cells to commit to the granulocyte-monocyte lineage [34]. Xie et al. showed overexpression of C/EBPα and C/EBPβ converted mature murine B cells into macrophages by suppressing PAX-5 expression [29]. Furthermore, studies using C/EBPα transgenic mice suggest that B cells are directly converted to other lineages through a biphenotypic intermediate cells rather than a two-step process of dedifferentiation and redifferentiation [34]. In support of a mechanism involving direct transdifferentiation, Feldman et al. showed loss of PAX-5 expression and up regulation of PU.1 and CEBPβ in eight cases of histiocytic-dendritic cell sarcomas derived from antecedent follicular lymphoma [12]. Since we do not know the expression pattern of PU.1, C/EBPα or C/EBPβ in these soft tissue tumors, their role in lineage transformation is unclear. However, PAX-5 was not expressed in the original PCM, therefore down regulation of PAX-5 cannot explain the development of monocytic-macrophage lineage. MUM-1, a transcription factor required for plasma cell differentiation [35], was expressed in the original plasma cell tumor but was not detected in the myeloid sarcomas (Figs. 1 and 3). Whether down-regulation of MUM-1 contributes to monocyte-macrophage transformation in plasma cell myeloma is unknown.

Limited data exist regarding the molecular genetics of transformed myeloid sarcoma; however several reports evaluating secondary HS suggest molecular complexity and heterogeneity. Similar to our case showing loss of CDKN2A/B, loss of CDKN2A has been reported in HS subsequent to B-lymphoblastic leukemia [15, 36]. BRAF V600E mutation has been reported in de novo HS [37] and HS following splenic marginal zone lymphoma [13] and hairy cell leukemia [38]. NGS analysis of the myeloid sarcoma in this case showed a clonal, non V600E activating mutation in BRAF. The BRAF mutation (G469A) is distinct from other variants identified in de novo HS, including BRAF F595 L, BRAF (G466R), BRAF (G464 V) and BRAF (N581S); however, as in this case, these BRAF variants are not mutually exclusive with activating RAS mutations [39, 40].

Like other cases of secondary HS [23], a clonal relationship between the primary PCM and secondary tumor in current case was confirmed by FISH and NGS analyses showing IGH-MAF gene rearrangement, and similar genomic alterations in KRAS, BRAF and MAK3K6. IGH-MAF translocation is present in 3–6% of PCM cases [41] and this molecular subtype often shows concurrent activating mutations in RAS-RAF and NF-kB signaling pathways [42]. In human cell lines, c-MAF promotes monocyte-macrophage differentiation through downreguation of CEBPα [43], suggesting a possible role of c-MAF in PCM phenotypic transformation.

In addition to shared clonal abnormalities, additional aberrancies were detected in the sarcoma tumor cells, including deletion of a subclone of BRAF (G466A), loss of TRAF3 R505 and new clonal mutations in NF1 and TNFAIP3 (Table 1). TRAF3 is a critical determinant of B cell survival and loss of function mutations in TRAF3 and TNFAIP3 are associated with B cell malignancies and PCM [44, 45]. NF1 mutation, a negative regulator of RAS signaling, has been reported in rare cases of plasma cell myeloma [4] and approximately 4% of acute myeloid leukemia [46], but not reported in HS.

It is uncertain whether the molecular switch from PCM to a myeloid lineage tumor involves direct transdifferentiation via transcription factor dysregulation as suggested for B cell lymphomas, or whether the myeloid lineage tumors arose from a distinct, neoplastic clone that expanded following chemotherapy [12, 15–18]. Regardless of the mechanism, NGS findings suggest a clonal relationship with clonal evolution and a possible role of NF1, TNFAIP3 and TRAF3 in myeloid transformation of plasma cell myeloma.

## Conclusion

To our knowledge, this is the first reported case of PCM transformation to a secondary tumor with monocyte-macrophage lineage. As in other reports, the response to chemotherapy and prognosis is poor with patients dying from progressive disease [18, 19, 23, 25, 26]. This study highlights the importance of molecular analysis to establish a clonal relationship in metachronous or synchronous tumors, as addressed by other reports [47, 48]. The findings of an additional mutation in RAS-BRAF signaling (NF1 mutation) and NF-kB activation (TNFAIP3) suggests multiple mechanisms contribute to lineage transformation.

## Abbreviations

FISH: Fluorescence in situ hybridization
HS: Histiocytic sarcoma
MPO: Myeloperoxidase
MS: Myeloid sarcoma
NGS: Next generation sequencing
PCM: Plasma cell myeloma
